# The relationship between upward social comparison and suicidal ideation: the mediating role of self-critical rumination and the moderating role of gender

**DOI:** 10.3389/fpsyg.2026.1816753

**Published:** 2026-06-10

**Authors:** Hui Li, Meng-ting Lin, Qin Jiang, Shu-wei Zhang

**Affiliations:** The School of Health, Fujian Medical University, Fuzhou, China

**Keywords:** college students, gender, self-critical rumination, suicidal ideation, upward social comparison

## Abstract

**Objective:**

This study aimed to investigate the mediating effect of self-critical rumination and the moderating effect of gender on the relationship between upward social comparison and suicidal ideation.

**Methods:**

A sample of 650 Chinese college students completed surveys including the Upward Social Comparison Scale, the Self-Rating Idea of Suicide Scale (SIOSS), and the Chinese Version of the Self-Critical Rumination Scale.

**Results:**

Upward social comparison was significantly and positively correlated with self-critical rumination and suicidal ideation (including dimensions: despair, optimism, and sleep factors). Self-critical rumination fully mediated the relationship between upward social comparison and suicidal ideation. Gender significantly moderated the pathway from selfcritical rumination to suicidal ideation, with a stronger positive association observed among female students.

**Conclusion:**

These findings clarify the mechanisms underlying the relationship between upward social comparison and suicidal ideation in college students and provide meaningful implications for intervention strategies aimed at reducing suicidal ideation. Specifically, suicide prevention efforts targeting female populations may benefit from placing greater emphasis on the regulation of self-critical rumination.

## 1 Introduction

Suicide represents a critical global public health and social issue. According to the World Health Organization, approximately 727,000 individuals worldwide die by suicide each year (“World Health Organization, 2025 World health statistics, 2025: monitoring health for the SDGs, sustainable development goals,” 2025). Among adolescents, the suicide rate has been rising at an alarming rate in recent years (Tian et al., 2023). As the initial stage in the continuum of suicidal behavior, suicidal ideation–which includes thoughts, desires, and other cognitive manifestations related to suicide–is considered the most sensitive predictor of subsequent suicidal acts (McHugh et al., 2019; Ying et al., 2024). A meta-analysis indicated that the prevalence of suicidal ideation among university students has reached 10.7% in recent years in China (Tan et al., 2022). Although only about 7.4% of those with suicidal ideation go on to attempt suicide (May and Klonsky, 2016), many of those who do not act on such thoughts may still remain trapped in a cycle of negative emotions (Jobes and Joiner, 2019). These individuals often struggle to break free from maladaptive behaviors and cognitive dissonance, which can ultimately impede personal growth and development. Therefore, investigating the mechanisms underlying suicidal ideation and implementing early interventions are of considerable importance for suicide prevention and the promotion of psychological well-being.

Existing research on suicide has largely focused on the role of situational risk factors (Witte et al., 2005). However, differences in suicidal ideation among individuals in similar situations suggest that cognitive-situational interactions, such as the evaluation, interpretation, and processing of negative situations, are key to understanding variations in the frequency and intensity of suicidal ideation (Klonsky et al., 2016; Wu and Yang, 2022; Wetherall et al., 2019a). In the increasingly competitive modern society, Social media makes information easily accessible. Combined with an innate drive for status, this fosters a growing prevalence of competitive mindsets in which the self is constructed and reinforced through social comparison (Aimah et al., 2024; Gilbert, 2020; Anderson et al., 2015). Assessing and responding to social rank information helps individuals cope with threat and stress. Upward social comparison is the tendency to compare oneself with better-off others when exposed to mixed social information (Gerber et al., 2018; Taylor et al., 1990). Gilbert proposed a three types of affect regulation system from an evolutionary perspective, noting that the perception of lower social status serves as a sensitive signal that activates an individual’s threat protection system (Gilbert, 2009, 2014, 2020). Furthermore, drawing on social rank theory, it explains that individuals possess an internal monitoring system for dynamic rank attention. Beyond objective low-status stimuli, this system generates subjective hierarchical signals through upward social comparison (Gilbert, 2020; Price et al., 2007; Gilbert et al., 1995), thereby activating the threat protection system and triggering negative experiences such as frustration, shame, and self-criticism, as well as cognitive biases that fixate on negative information (Gilbert, 2000; Cho and Ready, 2026; Gilbert and Miles, 2000). Previous studies based on the above theories have shown that low mood and submissive behavior are adaptive defensive strategies individuals employ to cope with frustrating situations such as blocked goals or status decline, choosing to yield and thereby signaling a de-escalation of competitive conflict (Gilbert, 2001; Price et al., 1994). However, persistent excessive upward social comparison may exacerbate depressive symptoms. This impairs the strategy’s function, blocks motivation to escape frustrating situations, creates a maladaptive dilemma, and raises suicide risk (Wetherall et al., 2019b; Nettle, 2004). Currently, a large body of research indicates that upward social comparison is closely associated with suicidal ideation, and a perceived decline in social status increases an individual’s suicidal ideation (Spitzer et al., 2023; Natasha et al., 2024; Dizon and Mendoza, 2023). Based on this, Hypothesis 1 is proposed: upward social comparison significantly and positively predicts suicidal ideation.

However, it remains unclear how upward social comparison translates into suicidal ideation via perceived lower social status. Social rank theory suggests that in competitive or conflict situations, upward social comparison leads individuals to perceive a disadvantaged position. Internal monitoring systems detect self-hostility. Self-criticism, as an involuntary subordinate strategy, alleviates threat from low-status signals through compliance and withdrawal. This reinforces threat protection system cognitive biases and intensifies focus on one’s own low status (Gilbert, 2020; Wetherall et al., 2019b). In the three types of affect regulation system, rumination focuses persistently on threat signals. This hinders escape strategies and creates entrapment. It also suppresses the drive system (resource seeking and goal pursuit) and the contentment system (security and satisfaction through self-compassion). This vicious cycle leads to suicidal ideation amid prolonged emotional dysregulation (Gilbert, 2009, 2014). It is worth noting that self-criticism and rumination are not entirely independent variables; previous studies have found that different types of rumination mediate the process by which self-criticism leads to suicidal ideation (O’Connor and Noyce, 2008). Specifically, self-critical rumination refers to maladaptive, repetitive thoughts of self-deprecation and self-attack in stressful situations such as failure, making mistakes, or failing to meet others’ or one’s own standards. It embodies the cognitive content of failure experiences and reflects a sense of entrapment, thereby providing a psychological foundation for the emergence of suicidal ideation (Tobias and Thomas, 2017; Smart et al., 2016). Existing research has confirmed a close association between self-critical rumination and emotional dysregulation, psychopathological symptoms, and suicide risk (Marian et al., 2021; Helena and Rodrigues, 2018; Kolubinski et al., 2020; Dehdari, 2025). In conjunction with the Integrated Motivational–Volitional (IMV) model of suicidal behavior (O’Connor and Kirtley, 2018), upward social comparison can be viewed as a background factor and vulnerability trait in the pre-motivational stage, which triggers the motivational stage by activating an individual’s perception of social status threats (Wetherall et al., 2019a). During the motivational stage, low-status signals trigger feelings of inferiority and failure. Internalizing this threat as self-critical rumination, a key cognitive mechanism of frustration and entrapment (Tobias and Thomas, 2017; Griffiths, 2014), reduces an individual’s cognitive flexibility and impairs their ability to effectively utilize psychological resources. Consequently, the individual feels unable to escape their current state of distress, ultimately leading to suicidal ideation (Bırni et al., 2025; Wetherall et al., 2019b). Based on this, Hypothesis 2 is proposed: self-critical rumination mediates the relationship between upward social comparison and suicidal ideation.

Gender significantly moderates the relationship between self-critical rumination and suicidal ideation. Females consistently report higher levels of both than males (Kou et al., 2018; Chen et al., 2024; Polanco-Roman et al., 2016). Previous studies show that rumination moderates suicidal ideation in women. Stress-related symptoms influence suicidal ideation via rumination only in females. In males, depressive symptoms primarily mediate this influence (Polanco-Roman et al., 2016). From the perspective of emotional coping strategies, this gender difference may be explained by females’ greater cognitive and affective susceptibility, which predisposes them to heightened emotional volatility and increased suicide risk (Kou et al., 2018; Chen et al., 2024). Divergent problem-solving approaches also contribute to this disparity. Response styles theory suggests that males tend to use distraction, whereas females tend to ruminate. Rumination narrows problem-solving options and intensifies entrapment, leading to higher overall rumination levels in females (Nolen-Hoeksema, 2003). Neurocognitive evidence links self-critical rumination in females to cognitive control processes in the left prefrontal cortex. This neural profile may underlie tendencies to fixate on negative information, engage in excessive counterfactual thinking, and show reduced proactive problem-solving (Allaert et al., 2025; Jens et al., 2021). Based on this, Hypothesis 3 is proposed: Gender moderates the second stage of the mediation pathway, specifically the relationship between self-critical rumination and suicidal ideation.

Theoretically, existing research has mainly examined bivariate associations among upward social comparison, rumination, and suicidal ideation. Discussions on specific cognitive pathways remain broad. First, generalized rumination includes adaptive reflection, but other subtypes do not focus on internal self-interaction. Only self-critical rumination clearly explains how self-threat from upward social comparison transforms into suicidal ideation (Diel et al., 2021). Second, upward social comparison is often described as a passive cognitive activity following stressful situations. However, even without stressors, individuals spontaneously engage in it to construct self-evaluation. Finally, both upward social comparison and self-critical rumination are maladaptive cognitive processing patterns triggered by comparing oneself to self-selected reference points in socially competitive contexts, they better explain the processing pathways leading to suicidal ideation in today’s highly competitive social environment. At the practical level, clarifying the core mediating role of self-critical rumination provides a new entry point for precise, targeted interventions and early identification of suicidal ideation. Compared with general rumination interventions, those targeting self-critical rumination can more specifically use psychotherapeutic methods such as compassion-focused therapy and self-compassion training. These methods address self-attack and self-criticism. They help establish appropriate self-pursuit goals and more effectively block pathways to suicidal ideation (Dehdari, 2025). Additionally, examining gender as a moderating variable can assist clinicians in developing differentiated intervention strategies for different gender groups.

**FIGURE 1:**
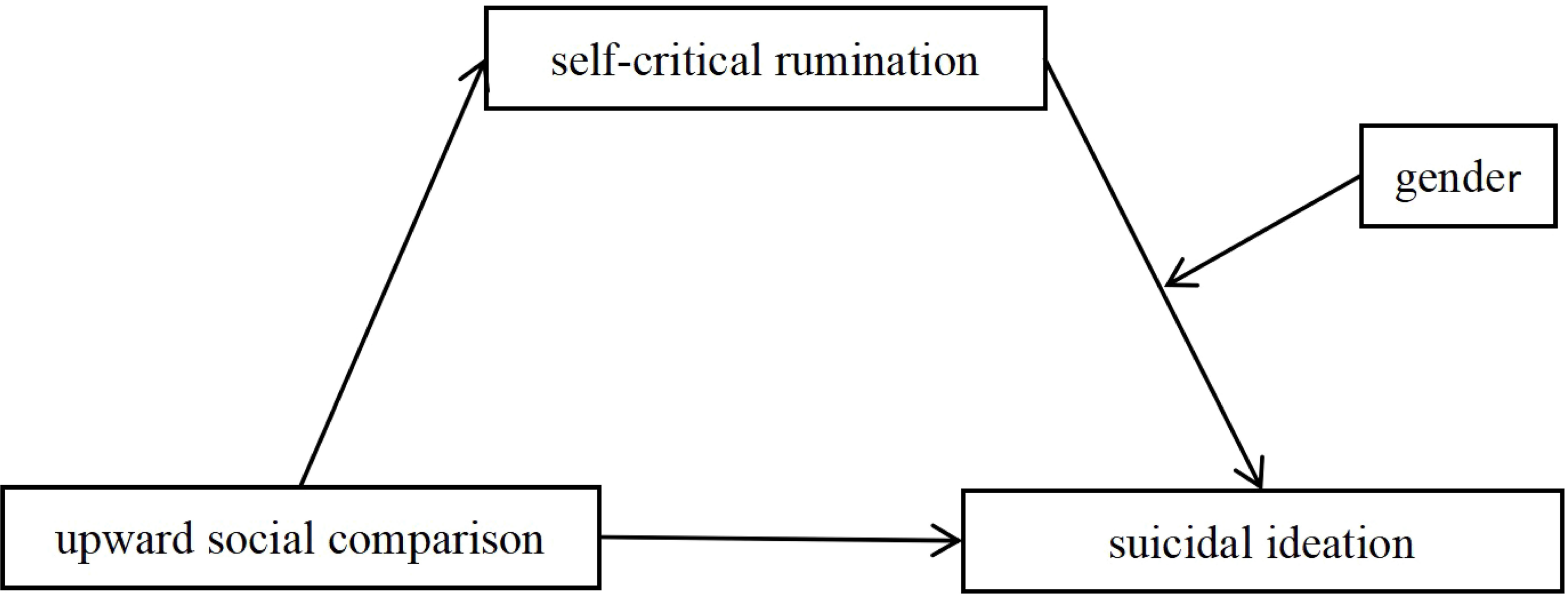
The proposed moderated-mediation model of the relationship between upward social comparison and suicidal ideation.

In summary, this study proposes a moderated mediation model to investigate the relationship between upward social comparison and suicidal ideation, as illustrated in Figure 1. Specifically, the model examines the mediating role of self-critical rumination in this association, along with the moderating effect of gender on the pathway from rumination to suicidal ideation. It is expected that the findings will contribute to a deeper understanding of the psychological mechanisms underlying suicidal ideation and offer novel implications for crisis intervention and clinical practice in suicide prevention.

## 2 Materials and methods

### 2.1 Participants

This study adopted a cross-sectional design to investigated a sample of Chinese university students. The research was conducted through an online survey, and ethical clearance was granted by the Biomedical Research Ethics Committee of Fujian Medical University. The principles of anonymity and confidentiality were strictly maintained throughout the data collection process. All participants provided informed consent before beginning the questionnaire. After excluding 98 unqualified questionnaires (13.11%) for failing attention checks (e.g., “For this question, please select ‘Strongly Agree”’), having an excessively short completion time (below three standard deviations), scoring high (≥4) on the concealment factor of the Suicidal Ideation Scale, and lacking informed consent, the final sample comprised 650 participants, resulting in an effective response rate of 86.89%. The final sample consisted of 174 males (26.8%) and 476 females (73.2%), with a mean age of 21.08 ± 2.12 years. Demographically, 323 students (49.7%) were from urban areas and 327 (50.3%) from rural areas. Seventy-eight participants (12.0%) came from single-parent families, while 572 (88.0%) were from non-single-parent families. Regarding sibling status, 265 participants (40.8%) were only children, and 385 (59.2%) had siblings.

### 2.2 Measures

#### 2.2.1 Subsubsection upward social comparison scale

The upward social comparison subscale was employed, which originated from the Iowa-Netherlands Comparison Orientation Measure developed by Gibbons and Buunk. This scale was later translated and adapted into Chinese by Bai et al. (2013). It comprises 6 items rated on a five-point Likert scale, from 1 (strongly disagree) to 5 (strongly agree). No items required reverse scoring, with higher total scores indicating a greater propensity for upward social comparison (Bai et al., 2013). In this study, the Cronbach’s α coefficient for this scale was 0.88.

#### 2.2.2 The Chinese version of the self-critical rumination scale

This study employed a Chinese version of the scale that was originally developed by Smart and subsequently localized and revised by Peng (2020). The revised scale comprises 10 items rated on a four-point Likert scale, with no reverse-scored items. Higher total scores reflect more severe self-critical rumination (Peng, 2020). To address non-normality in the data, robust maximum likelihood estimation (MLR) was employed. The results of the confirmatory factor analysis (CFA) indicated a good model fit: χ^2^/df = 4.05, TLI = 0.95, CFI = 0.96, RMSEA = 0.07, SRMR = 0.03, thereby supporting the scale’s construct validity among the university student sample. In this study, the Cronbach’s α coefficient for the scale was 0.91.

#### 2.2.3 The Self-Rating Idea of Suicide Scale (SIOSS)

The Chinese version of the Self-Rating Idea of Suicide Scale (SIOSS), developed by Xia et al. (2012), was administered. It contains 26 dichotomous items (0 or 1), which load onto four factors: despair, optimism, sleep, and concealment. The clinical cutoff for significant suicidal ideation is a total score of 12, higher scores indicate greater severity. Despair reflects hopelessness about the future and denial of self-worth, making death seem preferable to a hopeless life. Optimism is the belief that positive life experiences bring worth. Sleep refers to difficulty falling asleep, poor sleep quality, frequent waking, sometimes due to overthinking. The total score of suicidal ideation is the sum of three dimensions: despair, optimism, and sleep. The concealment factor is used only to screen for response distortion. It is not a substantive dimension of suicidal ideation and is excluded from subsequent analyses (Xia et al., 2012). In this study, the Cronbach’s α of total scale was 0.87.

### 2.3 Statistical analysis

Confirmatory factor analysis and multipath analysis using were conducted in Mplus (version 8.3). For other analyses, including common method bias testing, reliability analysis, descriptive statistics, correlation analysis, and independent samples *t*-tests, SPSS (version 27.0) was employed. Furthermore, the mediation and moderation models were tested using the PROCESS macro (version 4.1) developed by Hayes (2013).

## 3 Results

### 3.1 Participants common method deviation

Given that the data were collected exclusively through self-report measures, the potential for common method bias was acknowledged. To assess this possibility, Harman’s single-factor test was conducted. The results revealed eight factors with eigenvalues exceeding 1 prior to rotation. The first factor accounted for 26.675% of the total variance, which is below the critical threshold of 40%. This indicates that common method bias was not a serious concern in the present study.

### 3.2 Descriptive and correlation analyses

In this study, a total of 153 participants (23.54%) scored at or above the clinical cutoff (≥12) for suicidal ideation. Correlational analyses revealed that upward social comparison was positively associated with both self-critical rumination and suicidal ideation (including its three dimensions). Furthermore, self-critical rumination itself was positively correlated with suicidal ideation and all of its dimensions. Additionally, gender showed a significant, though weak, positive correlation with suicidal ideation and the despair factor, as shown in Table 1.

**TABLE 1:** Means, SD, and correlation matrix (*N* = 650).

Variable	*M*	*SD*	1	2	3	4	5	6	7
1. Upward social comparison	21.78	5.37	1.00	1.00	1.00	1.00	1.00	1.00	1.00
2. Self-critical rumination	25.95	6.42	0.53***						
3. Suicidal ideation	7.36	5.55	0.28***	0.55***					
4. Despair factor	4.43	3.54	0.30***	0.56***	0.96***				
5. Optimistic factor	1.09	1.46	0.19***	0.38***	0.83***	0.72***			
6. Sleep factor	1.84	1.36	0.16***	0.36***	0.70**	0.54***	0.42***		
7. Gender			−0.07	0.03	0.09*	0.11**	0.04	0.05	

*p < 0.05,

**p < 0.01 and

***p < 0.001.

Suicidal ideation and its despair factor exhibited significant variations by gender and geographical origin (*p* < 0.05). Specifically, female participants reported significantly higher levels of both suicidal ideation and hopelessness than males. Similarly, participants from rural areas reported significantly higher scores than those from urban areas. Furthermore, upward social comparison, self-critical rumination, suicidal ideation, and despair factor all showed significant differences depending on single-parent family status (*p* < 0.05). Participants from single-parent families scored higher across these variables compared to those from non-single-parent families.

### 3.3 The mediating role of self-critical rumination

Based on the results of demographic and statistical analyses, single-parent family status and geographical origin were included as control variables in the model. Given the gender imbalance in the sample, gender was also incorporated into the model as a control variable. All variables were standardized prior to analysis. The mediating role of self-critical rumination in the relationship between upward social comparison and suicidal ideation was examined using the Bootstrap method with Model 4 of the SPSS PROCESS macro developed by Hayes (2013).

The analysis revealed that upward social comparison significantly and positively predicted suicidal ideation (β = 0.29, SE = 0.04, *p* < 0.001), supporting Hypothesis 1. Furthermore, upward social comparison significantly and positively predicted self-critical rumination (β = 0.53, SE = 0.03, *p* < 0.001). When both upward social comparison and self-critical rumination were entered into the equation, the predictive effect of upward social comparison on suicidal ideation was no longer significant, while self-critical rumination significantly and positively predicted suicidal ideation (β = 0.54, SE = 0.04, *p* < 0.001), as shown in Table 2. Bootstrap analysis with 5,000 resamples further revealed a significant indirect effect of upward social comparison on suicidal ideation through self-critical rumination, with an effect size of 0.2885 and a 95% CI of (0.21, 0.36). In contrast, the direct effect was no longer significant, as shown in Table 3. These results indicate that self-critical rumination fully mediates the relationship between upward social comparison and suicidal ideation, thereby supporting Hypothesis 2.

**TABLE 2:** Testing the mediating model of self-critical rumination between upward social comparison and suicidal ideation.

Predictor variable	Suicidal ideation			Self-critical rumination			Suicidal ideation		
	β	*SE*	*t*	β	*SE*	*t*	β	*SE*	*t*
Gender	0.24	0.08	2.80**	0.15	0.08	1.97	0.16	0.07	2.11*
Geographic origin	−0.20	0.07	−2.72**	−0.06	0.07	−0.90	−0.17	0.07	−2.61*
Single-parent family status	−0.18	0.12	−1.55	−0.16	0.10	−1.58	−0.09	0.10	−0.90
Upward social comparison	0.29	0.04	7.69***	0.53	0.03	15.88***	−0.007	0.04	−0.19
Self-critical rumination		0.33			0.56		0.54	0.04	14.00***
R							0.54		
R^2^		0.11			0.29		0.31		
F		19.13***			65.67***		59.12***		

*p < 0.05,

**p < 0.01 and

***p < 0.001.

**TABLE 3:** The mediating role of self-critical rumination.

Effect	Effect size	BootSE	BootLLCI	BootULCI
Total effect	0.2885	0.04	0.21	0.36
Direct effect	−0.0007	0.04	−0.08	0.08
Indirect effect	0.2878	0.03	0.23	0.35

### 3.4 The moderating role of gender in the mediation model

The moderated mediation effect of gender was tested using Model 14 of the SPSS PROCESS macro. Based on prior demographic analyses, geographical origin and single-parent family status were included as control variables in all regression equations. All variables were standardized before analysis. The variance inflation factors for all predictor variables were below 1.5, indicating no substantial multicollinearity concerns.

Results showed a significant interaction effect between self-critical rumination and gender on suicidal ideation (β = 0.15, *t* = 2.06, *p* < 0.05), supporting Hypothesis 3 that gender moderates the relationship between self-critical rumination and suicidal ideation, as shown in Table 4.

**TABLE 4:** Test results for the moderated-mediated model.

Variable	Self-critical rumination			Suicidal ideation		
	β	*SE*	*t*	β	*SE*	*t*
Geographic origin	−0.07	0.07	−1.05	−0.16	0.06	−2.55*
Single-parent family status	−0.15	0.10	−1.50	−0.11	0.10	−1.12
Upward social comparison	0.53	0.03	15.75***	−0.004	0.04	−0.10
Gender			0.53	0.16	0.07	2.17*
Self-critical rumination				0.55	0.04	14.16***
Gender*self-critical rumination				0.15	0.07	2.06*
R					0.56	
R^2^			0.28		0.32	
F			85.88***		50.22***	

*p < 0.05,

**p < 0.01,

***p < 0.001.

**FIGURE 2:**
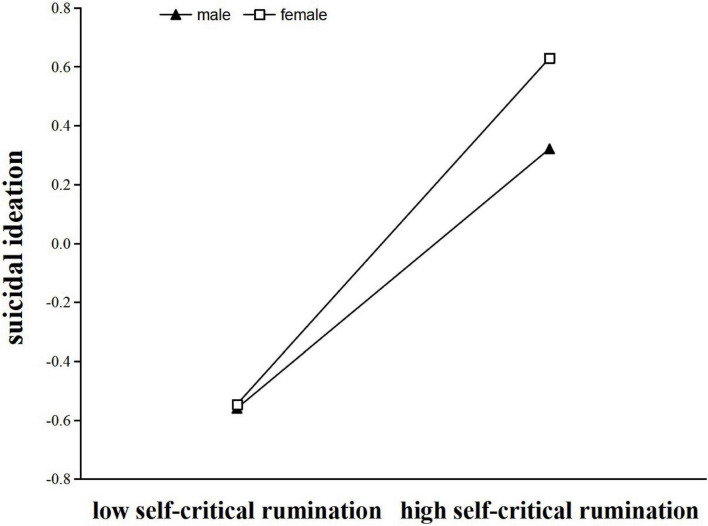
Moderating role of gender in the relationship between self-critical rumination and suicidal ideation.

To further interpret the interaction, a simple slope analysis was conducted. Self-critical rumination was categorized into high and low levels using ±1 SD from the mean, and the simple effects were tested separately for males and females. As shown in Figure 2, self-critical rumination significantly and positively predicted suicidal ideation for both male (Bsimple = 0.44, *t* = 7.05, *p* < 0.001) and female participants (Bsimple = 0.59, *t* = 13.17, *p* < 0.001). However, the relationship was significantly stronger among females, indicating that gender moderates the second stage of the mediation model–the path from self-critical rumination to suicidal ideation.

## 4 Discussion

Previous studies have focused on how negative emotions or events from upward social comparison affect suicidal ideation through rumination. However, they have not clarified how internal cognitive processing spontaneously transforms upward social comparison into suicidal ideation. That is, aggression and threats leading to suicidal ideation come not only from external sources but also from the self. Unlike transient self-criticism and general rumination, self-critical rumination serves as a key factor in this cognitive shift by continuously internalizing perceived hierarchical threat signals into persistent self-deprecation and self-attack, thereby driving individuals from feelings of frustration toward a sense of entrapment and ultimately suicidal ideation. From the perspective of internal self-interaction patterns, this study is the first to examine the relationship between upward social comparison and suicidal ideation among Chinese college students in a highly competitive environment. It also tested the mediating role of self-critical rumination and the moderating effect of gender. The results revealed that the effect of upward social comparison on suicidal ideation is fully mediated by self-critical rumination, and that gender moderates the latter half of the mediational pathway. These findings suggest that social upward comparison itself does not directly constitute a suicide risk but rather activates the individual’s threat perception system, thereby increasing suicide risk through the maladaptive coping mechanism of self-critical rumination. Future clinical interventions could target self-critical rumination specifically and employ compassion-focused therapy to disrupt this negative cycle.

### 4.1 Demographic analysis of variable

This study found that, in terms of gender, women exhibited significantly higher levels of suicidal ideation and despair, consistent with previous research (Wolfe et al., 2019). In terms of place of origin, rural participants scored higher on suicidal ideation, which may be related to impaired family functioning and negative cognitive structures resulting from their experience as left-behind children (Chen et al., 2024). Regarding family structure, participants from single-parent families scored significantly higher on measures such as upward social comparison, self-critical rumination, and suicidal ideation. This suggests that this group may exhibit cognitive vulnerability due to the unique nature of their environment, coupled with a lack of security and effective coping resources, which, through rumination, increases their risk of depression and suicide (Issar et al., 2017; Kou et al., 2018).

### 4.2 The relationship between upward social comparison and suicidal ideation

This study found that upward social comparison is significantly positively correlated with suicidal ideation and serves as a predictor of suicidal ideation, supporting Hypothesis 1 and consistent with previous research.

Social comparison theory suggests that individuals compare themselves with others to form a self-evaluation framework (Festinger, 1954). Previous research often focused on social comparison under external pressure and individual factors (Garcia et al., 2013). For instance, due to situational or resource uncertainty, individuals are forced to use social comparison to establish interpersonal ties. Failure to form such ties can evoke strong negative emotions (Xu and Li, 2024; Kulik and Mahler, 2000). However, from a social competition perspective, individuals possess competitive motivations. Even in non-stressful situations, they may actively select inappropriate targets and attributes for upward social comparison, producing self-threatening contrast effects (Xing and Yu, 2006; Taylor et al., 1990). Consequently, in the real-world environment characterized by the pervasive presence of social media, where curated and idealized information about advantaged others is ubiquitous, individuals frequently engage in upward social comparison along their chosen dimensions (Niu et al., 2016; Verduyn et al., 2020; Adrian and Johnson, 2022). In this situation, research confirms that upward social comparison is a core predictor of suicidal ideation and is positively correlated with positively correlated with feelings of frustration, depression, shame, and entrapment (Spitzer et al., 2023; Natasha et al., 2024; Zhao et al., 2022). The negative effects of upward social comparison are primarily manifested in emotional and attentional interference (Muller and Fayant, 2010). Individuals prone to upward social comparison process contrast information negatively. They focus more on superior, unattainable targets (Vogel et al., 2015). This process overactivates the threat protection system within Gilbert’s three types of affect regulation system, continually expanding the scope of social competition. This leads them to construct negative experiences, such as anxiety, depression, entrapment, and frustration, at their own level of inferiority. When the aggression stemming from these negative experiences is directed inward rather than outward, it reduces psychological resources, thereby triggering suicide risk (Vogel et al., 2015; Gilbert, 2000, 2020). Social rank theory further suggests that, during competition, individuals perceive themselves to be at a disadvantage through upward social comparison. To alleviate this conflict, the self-monitoring system continuously downplays one’s own abilities, thereby further reinforcing the cognitive biases generated by the threat protection system. This leads to an excessive focus on one’s own low-status signals, making individuals more prone to feelings of frustration and entrapment. These two key variables in the IMV model of suicide that contribute to suicidal ideation (Wetherall et al., 2019a,b; Gilbert, 2020). In summary, the association between upward social comparison and suicidal ideation is primarily influenced by negative cognitive patterns and a competitive mindset. When facing superior comparison targets, individuals can incorporate these targets into their self-representation. This activates assimilation-based social self-construction rather than using targets as a standard for self-evaluation. Such assimilation may enhance self-motivation and reduce suicide risk (Stapel and Koomen, 2001; Xing and Yu, 2006).

### 4.3 The mediating role of self-critical rumination

This study found that self-critical rumination fully mediates the relationship between upward social comparison and suicidal ideation. Specifically, upward social comparison increases the risk of suicidal ideation by activating self-critical rumination, a maladaptive cognitive process.

Goal-progression theory posits that when individuals encounter obstacles while pursuing higher-level goals, the accessibility of information related to unmet goals increases, making it easier for such information to be activated in the brain and leading to rumination (Martin and Tesser, 2006). As mentioned in the preceding discussion, the negative effects of upward social comparison are primarily manifested in emotional and attentional interference, and this interference is mediated through rumination (Davies, 2025). Within social rank systems, competitive outcomes give rise to dominant-subordinate relationships. When individuals perceive themselves to be in a disadvantaged position through upward social comparison, they adopt subordinate strategies, such as submission or flight, to express compliance toward higher-ranking individuals in order to avoid potential competitive harm (Gilbert, 2020; Allan and Gilbert, 1997). From this perspective, voluntary subordinate behavior may be adaptive, serving to avoid competitive conflict. However, when external dominance-subordination relationships are internalized at the self-level, that is, when one part of the self expresses criticism, aggression, and hatred, while another part feels suppressed and submissive, combined with increased accessibility to threatening information, the individual, through rumination, strives to escape this state or tendency. This evolves into involuntary subordinate strategies, intensifies entrapment, and leads to the passive choice of suicide as a coping strategy (Allan and Gilbert, 1997; Gilbert et al., 2004; Gilbert and Allan, 1994). Based on the IMV model of suicide, Wetherall argues that negative social comparison is a dependent variable of trait factors in the pre-motivational stage, influencing suicidal ideation through motivational variables such as frustration and feelings of entrapment (Wetherall et al., 2019a). In the motivational stage of the IMV model, rumination can function both as a mediator and as a moderator of the effects of frustration and feelings of entrapment on suicidal ideation (Dies et al., 2025). In summary, upward social comparison generates a sense of disparity between the self and the target, which activates an individual’s negative metacognition and persistent cognitive avoidance. Through self-critical rumination, this transforms feelings of frustration and disappointment into a perception of entrapment, making it impossible to maintain positive expectations and leading to the passive selection of suicidal ideation as a coping strategy.

At the same time, research has found that individuals who engage in high levels of self-critical rumination often exhibit greater maladaptive perfectionism, metacognitive disengagement, risk-related beliefs, and display more self-limiting behaviors (Fearn et al., 2022). This suggests that when pursuing high-level goals, they struggle to view their shortcomings rationally and are prone to self-denial, thereby reinforcing suicidal ideation. Although abandoning obstructed goals is an effective way to reduce self-critical rumination, individuals often find it struggle to let go of high-level comparative goal information. Therefore, reassessing one’s goals and committing to objectives more aligned with core values may be an effective strategy to avoid falling into a rumination cycle. Stimulating positive metacognition to initiate reflection rather than rumination, problem-solving can be effectively promoted (Marian et al., 2021; Kolubinski et al., 2017). On the other hand, self-critical rumination also desensitizes the internal sensory response to negative self-focus, making it difficult for individuals to regulate their emotions through bodily sensations and increasing their tendency to respond to psychological distress with extreme suicidal ideation (Young et al., 2021). Within the three types of affect regulation system, the contentment system can, through self-care, inhibit the activation of the threat and drive systems during frustration. Research has found that individuals with high psychological resilience can reduce their perceived stress when facing threats and challenges by suppressing the activation of the primary negative emotional system, avoiding maladaptive thought patterns such as rumination, and resisting their adverse effects (Hasanli et al., 2025). Thus, clinically, psychotherapies aimed at enhancing an individual’s emotional regulation abilities, such as compassion-focused therapy and other approaches, can effectively reduce self-critical rumination and prevent suicidal ideation by boosting psychological resilience, activating the self-compassion-based the contentment system, and breaking the vicious cycle (Dehdari, 2025; Gilbert et al., 2004; Fatollahzadeh et al., 2023).

### 4.4 The moderating role of gender

This study found that gender plays a moderating role in the latter half of the mediational model. Although the gender distribution of the sample was uneven, it was generally consistent with the epidemiological characteristics of suicidal ideation among Chinese college students: the proportion of females reporting suicidal ideation was significantly higher than that of males, with a gender ratio of approximately 2:1, indicating that the sample closely approximated the natural distribution in the real world (Yan and Gai, 2022). Due to the imbalance in gender ratios, further tests for invariance across groups were conducted. The results showed that the core pathway (self-critical rumination to suicidal ideation) did not exhibit significant differences between the male and female groups (*p* = 0.422), demonstrating that the mechanism identified in this study is stable and consistent across different gender groups. Therefore, although the proportion of females in the sample was relatively high, the findings still hold some reference value and generalizability for the male population.

In this study, the pathway through which self-critical rumination leads to suicidal ideation was stronger in females than in males. Consistent with previous findings, females’ suicidal ideation was more linked to rumination subtypes and depressive symptoms, whereas in males, depressive symptoms alone were a stronger predictor (Rich et al., 1992). Because females engage in more self-critical rumination, they are more prone to anxiety, depression, shame, and increased stress levels. This loss of self-worth leads to low self-esteem and greater susceptibility to psychopathological symptoms, which heightens suicidal ideation risk (Gold and Smout, 2024). Based on the IMV model, Vidal et al. (2024) found that females, due to a stronger sense of internal entrapment, are more likely to internalize emotional distress, leading to suicidal ideation; whereas males exhibit a stronger sense of entrapment in their external lives. Furthermore, gender differences in suicidal ideation may be related to multiple factors, including psychological vulnerability, social expectations, and gender roles. This divergence may stem from socialization processes that encourage females to internalize problems, while males are often socialized to externalize them. Consequently, females lack adequate psychological resources to break free from self-critical rumination, making them more prone to despair and leaving them with no alternative outlets other than suicidal ideation (Rich et al., 1992). Therefore, female college students’ self-critical rumination warrants targeted interventions such as self-compassion training. Furthermore, Allaert found that anodal tDCS of the left DLPFC temporarily reduces counterfactual thinking and regret in highly self-critical females. This form of neuromodulation technique holds promise for addressing the limitations of traditional psychological interventions (Jens et al., 2021).

### 4.5 Application value, limitations, and future research directions

A key finding of this study is that the impact of upward social comparison on suicidal ideation among college students is mediated by self-critical rumination, and that women are more sensitive to this mechanism. Based on this, the following intervention recommendations are proposed.

First, campus mental health education should identify social comparison and self-critical rumination. Activities such as class meetings and group counseling can guide students toward longitudinal self-growth comparisons and acceptance of shortcomings. Regular psychological assessments should also identify high suicide risk individuals. Second, cognitive restructuring training can correct irrational beliefs related to self-critical rumination. It may also complement Compassion-Focused Therapy and Acceptance and Commitment Group Therapy by activating self-compassion and empathy systems to break the rumination cycle (Dehdari, 2025). Notably, a digital therapy manual based on self-compassion can also effectively reduce students’ levels of self-critical rumination through the convenient medium of the internet (Kho et al., 2024). Finally, gender differences warrant attention. For females, rational emotive therapy and self-compassion interventions can release negative emotions and reduce self-directed aggression (Koróniová et al., 2020). In severe cases, neurostimulation techniques such as transcranial direct current stimulation may be considered. For males, the focus should be on problem-solving training to promote positive self-construction and reduce self-criticism stemming from feelings of frustration.

This study also has the following limitations: first, the cross-sectional design cannot establish strict causality. Future studies should use experimental designs to verify causal direction. Longitudinal approaches could also identify potential bidirectional relationships. Second, whereas self-critical rumination was assessed as a trait-level construct, suicidal ideation is known to exhibit short-term fluctuations. Subsequent investigations might therefore examine state-based measures of self-critical rumination as potentially more sensitive predictors of transient suicidal ideation. Third, this study focuses on risk factors for suicidal ideation and does not include an examination of protective factors such as psychological resilience and social support. Future research could explore, based on the three types of affect regulation system, whether activation of the contentment system can serve as a moderating factor in reducing suicide risk. Fourth, the sample had a gender imbalance. Given the small number of males, gender-specific results require caution. Supplementary analyses support a consistent cross-gender mechanism, but future validation in a more balanced sample is needed. These findings are preliminary and exploratory, not definitive. Future studies should recruit larger, gender-balanced samples or use stratified sampling to verify robustness of the present findings.

## 5 Conclusion

In summary, upward social comparison was found to be a positive predictor of suicidal ideation, with this relationship being fully mediated by self-critical rumination. Furthermore, gender moderated the second stage of this mediation pathway, as the association between self-critical rumination and suicidal ideation was significantly stronger among female participants than among males. These findings highlight self-critical rumination as a precise target for clinical intervention aimed at reducing suicidal ideation. Specifically, they suggest that suicide prevention efforts targeting female populations may benefit from placing greater emphasis on the regulation of self-critical rumination.

## Data Availability

The original contributions presented in this study are included in the article, further inquiries can be directed to the corresponding authors.
